# Radiological Clinical Practice Guidelines Published in the Last Decade: A Bibliometric Analysis

**DOI:** 10.5334/jbsr.1764

**Published:** 2019-06-28

**Authors:** Su Jin Hong, Dae Young Yoon, Kyoung Ja Lim, Ji Yoon Moon, Soo Jeong Yoon, Young Lan Seo, Eun Joo Yun

**Affiliations:** 1 Kangdong Seong-Sim Hospital, Hallym University College of Medicine, KR

**Keywords:** bibliometrics, publication, radiologists, clinical practice guideline, radiologic guideline, comprehensive review

## Abstract

**Objectives:**

To provide a comprehensive review of radiological clinical practice guidelines (CPGs) and to establish their characteristics and impact in the field of radiology.

**Material and Methods:**

A MEDLINE search was performed for CPGs in which at least half of the authors were from the radiology or imaging department. The following information was extracted from each CPG: year of publication, journal, provider, number of authors, number of pages, number of references, collaboration, country of origin, radiological subspecialty, imaging modality used, topic, source of funding, and number and pattern of citations.

**Results:**

In total, 120 radiological CPGs published between July 2006 and June 2016 were identified. One hundred nine (90.8%) radiological CPGs were published in radiology journals, 96 (80.0%) were provided by the scientific community, 108 (90.0%) were collaborative studies, 64 (53.3%) originated from the United States, 36 (30.0%) were concerned with the field of vascular/interventional radiology, 38 (31.7%) used combined imaging techniques, 52 (43.3%) were focused on interpretation and management, and 118 (98.4%) were not funded. Radiological CPGs included a median of 8 authors, 9 pages, and 49 references. The median number of citations and annual citations were 18 (range, 0–540) and 3.5 (range, 0–75.6), respectively.

**Conclusion:**

Our study presents several interesting insights into the characteristics and impact of radiological CPGs.

## Introduction

A clinical practice guideline (CPG) is a systematic statement that presents the most appropriate recommendations to assist health care practitioners in making diagnostic and therapeutic decisions in specific clinical circumstances [1]. Most CPGs are based on evidence-based medicine and systematic literature review of published studies.

Radiology is a technology-based specialty of medicine that uses medical imaging modalities to diagnose and treat diseases and disorders. Owing to technical and technological advances, the field of radiology has rapidly developed in recent years and many important scientific observations have been made. However, the adoption of evidence-based medicine in radiology has been delayed because imaging technology has improved so rapidly that it has become difficult to evaluate existing imaging modalities, especially when long-term clinical data are required [2,3]. However, since the inception of the evidence-based practice approach in radiology, the number of published systematic reviews has continuously increased in recent times [4]. Although it can be hypothesized that this environment may stimulate the development of evidence-based CPGs to help radiologists in their activities, no previous study to our knowledge has evaluated radiological CPGs.

Therefore, the purpose of this bibliometric analysis was to provide a comprehensive review of radiological CPGs and to establish their characteristics and impact in the field of radiology.

## Material and Methods

This bibliometric analysis of a publicly available database was exempt from Institutional Review Board approval. Two reviewers independently reviewed the full text of articles to identify eligible studies and extract data. Discrepancies were resolved by arbitration with a third reviewer.

### Search strategy

The National Library of Medicine’s MEDLINE database was searched via the PubMed (http://www.ncbi.nlm.nih.gov/pubmed/) in July 2017 to identify all radiological CPGs published between July 2006 and June 2016, which allowed for a citation window of at least one full year. For the purposes of this study, “a radiological CPG” was defined as a CPG in which at least half of the authors were affiliated with a radiology or imaging department.

We performed a search by typing “radiol*” or “imag*” in the author affiliation field and “guideline” in the MEDLINE-defined publication type. The terms radiol* and imag* were chosen in order to widen our search to authors with a variety of addresses (e.g., Department of Neuroradiology, Department of Pediatric Radiology, Imaging Center, or Department of Medical Imaging) and to exclude authors affiliated to the “Department of Nuclear Medicine” and “Department of Radiation Oncology” because these departments are separate from the Department of Radiology in most countries.

### Inclusion and exclusion criteria

We identified 298 articles by employing the abovementioned search approach. Among them, 178 were excluded from the analysis because of the following reasons: 1) articles in which at least half of the authors were not affiliated to a radiology or imaging department (n = 147), 2) articles focusing on the evaluation of CPG (review, appraisal, validation, addendum, commentary, opinion, and letter) (n = 10), 3) articles published in non-English journals (n = 8), 4) duplicate publications of the same CPG (n = 7), and 5) articles for which the full text was not available either online or in print (n = 6). Finally, the remaining 120 CPGs were included in the study.

### Data extraction and analysis

To establish the characteristics and impact of CPGs in the field of radiology, the following data were extracted from each article: 1) journal, 2) impact factor (IF) of the journal, 3) subject category of the journal (radiology, other specialties, or multidisciplinary), 4) provider (personal, study group, or scientific community), 5) number of authors, 6) number of pages, 7) number of references, 8) collaboration (none, interdepartmental, multi-institutional, or international), 9) country of origin, 10) radiological subspecialty (abdominal, breast, cardiac, chest, genitourinary, musculoskeletal [including spine], neuroradiology/head and neck, pediatric, thyroid, vascular/interventional, or miscellaneous [not conforming to one of the abovementioned categories, including physics, basic science, whole-body imaging, contrast media, and radiation protection]), 11) imaging modality used (conventional radiography, ultrasonography, computed tomography [CT], magnetic resonance imaging [MRI], angiography, mammography, nuclear medicine, combined [more than one imaging modality used], or miscellaneous [not conforming to one of the abovementioned categories]), 12) topic, 13) source of funding (none, government, private, industry, or multiple), 14) number of total citations, 15) number of annual citations (calculated using the total number of citations over the number of years and months since publication), and 16) citation pattern of 91 radiological CPGs in the five years after publication.

The IF and subject category of each journal were retrieved from the Journal Citation Reports (Thomson Reuters, New York, NY) for the year 2016. Journals that fell into more than one category were manually reviewed and assigned to only one category based on the title of the journal and information contained on the journal’s website. For articles arising from a collaboration involving more than one country, the country of the first author was considered the country of origin of the paper. If the first author had affiliations in more than one country or was a group name, the country of the corresponding author was considered the country of origin. CPGs for imaging-guided therapeutic procedures were assigned to the vascular/interventional subspecialty, while CPGs for imaging-guided needle biopsies were included in the appropriate organ or system subspecialty. The numbers of total citations and annual citations of CPGs from publication to the time of the search were identified using the Web of Science (Thomson Reuters) on June 30, 2017. In addition, to characterize citation patterns over time, we calculated the mean number of citations of radiological CPGs for publication and at the first, second, third, fourth, and fifth years after publication.

The present study adopted descriptive statistics by means of scientometric analysis. Data were analyzed using Microsoft Excel (Microsoft, Redmond, WA, USA), to determine their frequency (absolute and relative), central tendency (median), and dispersion (range). For trend analyses, linear regression test was used for each variable. Statistical analysis was performed using IBM SPSS Statistics for Windows/Macintosh (Version 20.0; IBM Corp., Armonk, NY, USA), and a *p*-value of < 0.05 was considered statistically significant.

## Results

The journal that published the highest number of radiological CPGs was the *Journal of Vascular and Interventional Radiology* (n = 34), followed by *CardioVascular and Interventional Radiology* (n = 12) and *European Radiology* (n = 9) (Table 1). One hundred nine (90.8%) radiological CPGs were published in radiology journals. The United States was the most productive country with 64 (53.3%) publications on radiological CPGs. The United Kingdom, Canada, Germany, and Italy also contributed greatly by publishing more than five CPGs (Table 2).

**Table 1:** Top 10 journals with the highest number of radiological clinical practice guidelines.

Rank	Journal	Journal IF 2016	No. of Articles

1	*Journal of Vascular and Interventional Radiology*	2.78	34
2	*CardioVascular and Interventional Radiology*	2.19	12
3	*European Radiology*	3.97	9
4	*Journal of the American College of Radiology*	2.99	8
5	*Canadian Association of Radiologists*	1.27	6
6	*Pediatric Radiology*	1.47	5
7	*American Journal of Roentgenology*	2.78	3
7	*Korean Journal of Radiology*	2.16	3
7	*International Journal of Cardiovascular Imaging*	1.89	3
7	*Japanese Journal of Radiology*	0.98	3
7	*European Journal of Ultrasound*	0.91	3

*Note*: IF = impact factor.

**Table 2:** Top 10 countries by the number of radiological clinical practice guidelines.

Rank	Country	No. of articles (%)

1	United States	64 (53.3)
2	United Kingdom*	10 (8.3)
3	Canada	7 (5.8)
4	Germany	6 (5.0)
5	Italy	5 (4.2)
6	Austria	4 (3.3)
6	The Netherlands	4 (3.3)
8	France	3 (2.5)
8	Japan	3 (2.5)
8	South Korea	3 (2.5)

*Note*: The percentages do not add up to 100% because the shares of other countries are not included. * Includes articles originating from England, Scotland, Wales, and Northern Ireland.

Ninety-six (80.0%) radiological CPGs were provided by the scientific community, 108 (90.0%) were collaborative studies, 36 (30.0%) were concerned with the field of vascular/interventional radiology, 38 (31.7%) used combined imaging techniques, 52 (43.3%) were focused on interpretation and management, and 118 (98.4%) were not funded (Table 3). The median number of authors, pages, and references in radiological CPGs was 8 (mean, 9.2 ± 6.7; range, 1–45), 9 (mean, 10.2 ± 6.3; range, 2–35), and 49 (mean, 62.0 ± 54.9; range, 0–278), respectively.

**Table 3:** Characteristics of 120 radiological clinical practice guidelines.

	No. of Articles (%)

Subject category of the journal	
Radiology	109 (90.8)
Other specialties	11 (9.2)
Provider	
Scientific community	96 (80)
Study group	17 (14.1)
Personal	7 (5.8)
Collaboration	
Multi-institutional	75 (62.5)
International	31 (25.8)
Interdepartmental	2 (1.7)
None	12 (10)
Radiological subspecialty	
Vascular/interventional	36 (30)
Abdominal	15 (12.5)
Cardiac	13 (10.8)
Neuroradiology/head and neck	9 (7.5)
Pediatric	8 (6.7)
Musculoskeletal	6 (5)
Chest	5 (4.2)
Genitourinary	5 (4.2)
Miscellaneous	23 (19.2)
Imaging modality used	
Angiography	22 (18.3)
Computed tomography	15 (12.5)
Magnetic resonance imaging	12 (10)
Ultrasonography	8 (6.7)
Nuclear medicine	2 (1.7)
Conventional radiography	1 (0.8)
Combined	38 (31.7)
Others	22 (18.3)
Topic	
Interpretation and management	52 (43.3)
Intervention	46 (38.3)
Technique and protocol	9 (7.5)
Contrast media	7 (5.8)
Radiation protection	5 (4.2)
Others	1 (0.8)
Source of funding	
Government	2 (1.6)
Private	0 (0)
Industry	0 (0)
None	118 (98.4)

The median number of total and annual citations was 18 (mean, 44.1 ± 73.9; range, 0–540) and 3.5 (mean, 7.2 ± 11.2; range, 0–75.6), respectively. The annual number of citations of radiological CPGs reached a peak in the fourth year after publication and decreased thereafter (Table 4). Supplement 1 and 2 lists the top 10 radiological CPGs with the highest numbers of total and annual citations, respectively.

**Table 4:** Citation pattern of 91 radiological clinical practice guidelines in the 5 years after publication.

Mean No. of Citations by Year after Publication				

≤First Year	Second Year	Third Year	Fourth Year	Fifth Year

1.84	6.31	8.19	10.11	8.98

## Discussion

CPGs are developed to offer a standard tool for health care practitioners to make conscious and judicious decisions about appropriate management of patients. In other words, radiological CPGs are designed to help radiologists, medical technicians, and nurses working in the department of radiology “do the right thing” in a specific clinical circumstance [5]. CPGs are not only based on clear-cut data published in the medical literature, but also on the experiences of experts, consensus among members of the scientific community, public health policies, and budgetary limitations [6]. Although CPGs may be important in standardized medical behavior, they may lead to harmful choices in the case of individual patients if they do not really advocate the best options for patients because of scientific uncertainties, biases in guideline development, and patient heterogeneity [7].

Overall, 120 radiological CPGs were published during the last 10 years (12 CPGs per year). This number of radiological CPGs may reflect a recent awareness of the importance of CPGs in the field of radiology. In addition, the appearance of new scientific communities in the field of radiology may have contributed to this popularity. Scientific communities could be labeled as the most efficient environment for production and utilization of CPGs. As a result, many scientific communities in the field of radiology had a great role in developing radiological CPGs, and they contributed to the publication of 80% of CPGs.

Despite these achievements, two disappointing aspects in the publication of radiological CPGs should be noted. First, although 90% of the radiological CPGs were the result of collaboration at various levels, only 25.8% of CPGs were created by international collaboration. This low proportion of international collaboration may suggest the lack of cooperation between national scientific communities. It would be reasonable to assume that more international collaboration would lead to more globally standardized CPGs. Second, only 1.6% of radiological CPGs received funding. This funding rate is markedly lower than that previously reported for interventional radiology (23.0%) [8] and for general radiology (26.9%) [9]. The importance of research funding and its positive association with the quality of research has been well established [10].

There was a large variation in the rate of publication of CPGs between different imaging subspecialties. The most active subspecialties were vascular and interventional radiology, accounting for 30.0% of radiological CPGs. Vascular and interventional radiology is one of the most rapidly developing fields of medicine in which minimally invasive procedures are performed using image guidance for the diagnosis and treatment of various diseases. This progress was mainly attributable to continuing advances in interventional techniques, imaging instruments, and various medical devices. Therefore, many updated CPGs were necessary for effective and safe therapeutic procedures.

When analyzing the first authors’ addresses, more than half of the radiological CPGs were from the United States. This finding is unsurprising given the large size of the health care system and the well-developed networks of scientists in the United States. In addition, the United States and United Kingdom were the two leading publishers of CPGs, accounting for 61.7% of all articles. In a previous study [9], the United States and United Kingdom published 48.5% of original articles in the AJR and Radiology between 2001 and 2010. This difference suggests that these two countries have greater ability to produce CPGs than their overall research productivity. Both the United States and United Kingdom are English-speaking countries, and journals with high IFs publish articles written only in English. Thus, authors from these nations can write long CPGs more clearly and concisely than do their non–English-speaking colleagues [11].

The number of citations since publication is a widely accepted index for determining the impact of a scientific article on its field [12]. We also assessed the pattern of citations of radiological CPGs over a 5-year window after publication and found that the mean number of annual citations reached a peak in the fourth year after publication and showed a slowly decreasing pattern thereafter. This finding is different from that of a recent citation analysis of imaging literature [13], which reported that the mean number of annual citations of review articles in imaging literature was highest during the third year after publication. In addition, radiological CPGs have approximately 1.5-fold to 2.5-fold higher number of annual citations than do review articles between the second and fifth years after publication [13]. A higher number and longer duration of increase of annual citations for CPGs than for review articles reflect the strong scientific impact and high citation power of radiological CPGs.

This bibliometric analysis has several limitations. First, the search process was limited to journals indexed in the MEDLINE database. Consequently, radiological CPGs published in other respected journals, including many local journals, were missed by the methodology used in this study. Second, it should be kept in mind that not all CPGs are eventually published. We did not search websites of all organizations and scientific communities potentially developing radiological CPGs. Therefore, non-published radiological CPGs were not included in our data. Finally, we included articles classified as “guidelines” in the MEDLINE-defined publication type. Thus, the numbers of CPGs in our study may be inaccurate if some articles were misclassified in the MEDLINE database.

In conclusion, this bibliometric analysis revealed several interesting insights into the characteristics and impact of radiological CPGs.

## Additional Files

The additional files for this article can be found as follows:

10.5334/jbsr.1764.s1 Supplement 1. The top 10 radiological clinical practice guidelines with the highest number of total citations.

10.5334/jbsr.1764.s2 Supplement 2. The top 10 radiological clinical practice guidelines with the highest number of annual citations.
