# Supplementary material for: Radiological Clinical Practice Guidelines Published in the Last Decade: A Bibliometric Analysis
Source: J Belg Soc Radiol. 2019 Jun 28;103(1):37. doi: 10.5334/jbsr.1764 (PMC6598615; doi:10.5334/jbsr.1764)
Supplement: Supplement 1. — The top 10 radiological clinical practice guidelines with the highest number of total citations. [file jbsr-103-1-1764-s1.pdf]

Supplement 1. The top 10 radiological clinical practice guidelines with the highest number of total citations.

Rank	Article	No. of Citations
1	Claudon M, et al. Guidelines and good clinical practice recommendations for contrast enhanced ultrasound (CEUS) – update 2008. Part 1: Basic principles and technology. Ultraschall Med. 2008; 29:28–44.	540
2	Bamber J, et al. EFSUMB guidelines and recommendations on the clinical use of ultrasound elastography. Part 1: Basic principles and technology. Ultraschall Med. 2013; 34:169–84.	315
3	Kramer CM, et al. Standardized cardiovascular magnetic resonance imaging (CMR) protocols, society for cardiovascular magnetic resonance: Board of trustees task force on standardized protocols. J Cardiovasc Magn Reson. 2008; 10:35.	307
4	Claudon M, et al. Guidelines and good clinical practice recommendations for contrast enhanced ultrasound (CEUS) in the liver—update 2012: A WFUMB-EFSUMB initiative in cooperation with representatives of AFSUMB, AIUM, ASUM, FLAUS and ICUS. Ultraschall Med. 2013; 34:11–29.	209
5	Chen MM, et al. Guidelines for computed tomography and magnetic resonance imaging use during pregnancy and lactation. Obstet	207

	Gynecol. 2008; 112:333–40.	
6	Moon WJ, et al. Ultrasonography and the ultrasound-based management of thyroid nodules: Consensus statement and recommendations. Korean J Radiol. 2011; 12:1–14.	189
7	Stecker MS, et al. Guidelines for patient radiation dose management. J Vasc Interv Radiol. 2009; 20:S263–73.	171
8	Van Der Molen AJ, et al. CT urography: Definition, indications and techniques. A guideline for clinical practice. Eur Radiol. 2008; 18:4–17.	166
9	Strauss KJ, et al. Image gently: Ten steps you can take to optimize image quality and lower CT dose for pediatric patients. AJR Am J Roentgenol. 2010; 194:868–73.	151
10	Kanal E, et al. ACR guidance document on MR safe practices: 2013. J Magn Reson Imaging. 2013; 37:501–30.	149
