# Supplementary material for: Radiological Clinical Practice Guidelines Published in the Last Decade: A Bibliometric Analysis
Source: J Belg Soc Radiol. 2019 Jun 28;103(1):37. doi: 10.5334/jbsr.1764 (PMC6598615; doi:10.5334/jbsr.1764)
Supplement: Supplement 2. — The top 10 radiological clinical practice guidelines with the highest number of annual citations. [file jbsr-103-1-1764-s2.pdf]

Supplement 2. The top 10 radiological clinical practice guidelines with the highest number of annual citations.

Rank	Article	No. of Annual Citations
1	Bamber J, et al. EFSUMB guidelines and recommendations on the clinical use of ultrasound elastography. Part 1: Basic principles and technology. Ultraschall Med. 2013; 34:169–84.	75.6
2	Claudon M, et al. Guidelines and good clinical practice recommendations for contrast enhanced ultrasound (CEUS) - update 2008. Part 1: Basic principles and technology. Ultraschall Med. 2008; 29:28–44.	57.9
3	Claudon M, et al. Guidelines and good clinical practice recommendations for contrast enhanced ultrasound (CEUS) in the liver--update 2012: A WFUMB-EFSUMB initiative in cooperation with representatives of AFSUMB, AIUM, ASUM, FLAUS and ICUS. Ultraschall Med. 2013; 34:11–29.	48.2
4	Kanal E, et al. ACR guidance document on MR safe practices: 2013. J Magn Reson Imaging. 2013; 37:501–30.	35.1
5	Kramer CM, et al. Standardized cardiovascular magnetic resonance imaging (CMR) protocols, society for cardiovascular magnetic resonance: Board of trustees task force on standardized	34.4

	protocols. J Cardiovasc Magn Reson. 2008; 10:35.	
6	Moon WJ, et al. Ultrasonography and the ultrasound-based management of thyroid nodules: Consensus statement and recommendations. Korean J Radiol. 2011; 12:1–14.	29.5
7	Patel IJ, et al. Consensus guidelines for periprocedural management of coagulation status and hemostasis risk in percutaneous image-guided interventions. J Vasc Interv Radiol. 2012; 23:727–36.	26.2
8	Chen MM, et al. Guidelines for computed tomography and magnetic resonance imaging use during pregnancy and lactation. Obstet Gynecol. 2008; 112:333–40.	23.4
9	Stecker MS, et al. Guidelines for patient radiation dose management. J Vasc Interv Radiol. 2009; 20:S263–73.	21.4
10	Strauss KJ, et al. Image gently: Ten steps you can take to optimize image quality and lower CT dose for pediatric patients. AJR Am J Roentgenol. 2010; 194:868–73.	21.1
